# The predictive value of cervical shear wave elastography in the outcome of labor induction

**DOI:** 10.1111/aogs.13706

**Published:** 2019-11-05

**Authors:** Jing Lu, Yvonne Kwun Yue Cheng, Sin Yee Stella Ho, Daljit Singh Sahota, L. L. Hui, Liona C. Poon, Tak Yeung Leung

**Affiliations:** ^1^ Department of Obstetrics and Gynecology Prince of Wales Hospital The Chinese University of Hong Kong Hong Kong SAR China; ^2^ Department of Obstetrics and Gynecology The First Affiliated Hospital of Xiamen University Xiamen China; ^3^ Department of Imaging and Interventional Radiology Prince of Wales Hospital The Chinese University of Hong Kong Hong Kong SAR China; ^4^ Department of Pediatrics Prince of Wales Hospital The Chinese University of Hong Kong Hong Kong SAR China

**Keywords:** angle of progression, Bishop score, cervical length, induction of labor, posterior cervical angle, prediction, shear wave elastography

## Abstract

**Introduction:**

Bishop score, the traditional method to assess cervical condition, is not a promising predictive tool of the outcome of labor induction. As an objective assessment tool, many cervical ultrasound measurements have been proposed to represent the individual components of the Bishop score, but none of them can measure the cervical stiffness. Cervical shear wave elastography is a novel tool to assess the cervical stiffness quantitatively.

**Material and methods:**

A total of 475 women who required labor induction were studied prospectively. Prior to routine digital assessment of the Bishop score, transvaginal sonographic measurement of cervical length, posterior cervical angle, angle of progression and shear wave elastography was performed. Shear wave elastography measurement was made at the inner, middle and outer regions of the cervix to assess homogeneity. Association of labor induction outcomes including the overall cesarean section and subgroups of cesarean section for failure to enter active phase, with cervical sonographic parameters and the Bishop score, were assessed using multivariate regression analyses. The predictive accuracy of the outcomes using models based on ultrasound measurement and the Bishop score was compared using the area under the receiver‐operating characteristics curves.

**Results:**

Among 475 women, 82 (17.3%) required cesarean section. Shear wave elasticity was significantly higher in the inner cervical region than in other regions, indicating a greater stiffness (*P *< 0.001). Both inner cervical shear wave elasticity and cervical length were independent predictors of overall cesarean section (respective adjusted odds ratio [95% CI] 1.338 [1.001‐1.598] and 1.717 [1.077‐1.663]) and cesarean section for failure to enter active phase (respective adjusted odds ratio [95% CI] 1.689 [1.234‐2.311] and 2.556 [1.462‐4.467]), after adjusting for other covariates. Outcome prediction models using inner cervical shear wave elasticity and cervical length, had increased area under curve compared with models using the Bishop score (0.888 vs 0.819, *P *= 0.009).

**Conclusions:**

The cervix is not a homogenous structure, with the inner cervix having the highest stiffness, which is an independent predictor of overall cesarean section, and specifically for those indicated because of failure to enter active phase. Models based on shear wave elastography and cervical length had higher predictive accuracy than models based on the Bishop score.

**Abbreviations**
AOP: angle of progression
AOR: adjusted odds ratio
AUC: area under curve
BMI: body mass index
BS: Bishop score
CS: cesarean section
IOL: induction of labor
PCA: posterior cervical angle
ROC: receiver‐operating characteristics
SWE: shear wave elastography

**Key message**

Shear wave elastography is a useful predictor of cesarean section. The combination of sonographic cervical length and elastography is superior to the Bishop score in the prediction of the outcome of labor induction.

## 1 INTRODUCTION

Approximately one in five inductions of labor (IOL) results in an emergency cesarean section (CS) due to failure to reach active phase or labor, failure to progress beyond the active phase or fetal distress.^1^ The expected outcome and management of IOL has traditionally been based on vaginal digital assessment of the cervix to assess the Bishop Score (BS).^2^ However, the BS has been shown to be subjective^3^ and has relatively low predictive performance.^4^ Recent research has therefore focused on the use of ultrasound for more objective assessment of individual components of BS. Besides cervical length, posterior cervical angle (PCA)^5^ and angle of progression (AOP)^6^ can be measured sonographically to reflect the cervical position and fetal head descent, respectively. Although some studies have shown that these ultrasonic measurements are superior to digital assessment, their predictive values remain suboptimal for clinical use.^7^, ^8^ One main reason for this is the inability to measure cervical consistency, a major component of BS, using conventional ultrasound technology. Hence strain‐based sonoelastography has also been investigated for measuring uterine cervical stiffness. However, this requires human movements on the ultrasound probe to generate a ‘stress’ on the target tissue. It is limited to measuring relative strain of the target tissue in comparison with its adjacent tissues, but the cervix lacks good surrounding tissues to act as reference.^9^ Several small‐cohort studies have conflicting results regarding the predictive value of strain‐based elastography.^10^, ^11^, ^12^, ^13^ In contrast, shear wave elastrography (SWE) uses ultrasound pulses to generate shear waves across a target tissue, and the shear wave velocity (‘v’) correlates to the tissue stiffness. Young's modulus (E) in kPa can be estimated using the formula E ≅ 3ρv^2^, where ρ is the density of the tissue (kg/m^3^), which is assumed to be constant.^14^ SWE assessment of the cervix has recently been reported to have good intra‐ and interobserver reproducibility.^15^ Cervical stiffness using SWE was also shown to decrease with gestational age.^15^ Yet its value in predicting IOL outcome is unknown. Therefore the aim of the present study is to evaluate whether cervical SWE can improve the predictive performance of the outcome of IOL when combined with other ultrasound‐based assessments as compared with BS alone.

## 2 MATERIAL AND METHODS

### 2.1 Study design

This was a prospective observational study conducted between September 2015 and November 2017. Women admitted for IOL were invited to participate. The inclusion criteria were: (1) Chinese women carrying singleton pregnancies; (2) ≥37 gestational weeks; (3) vertex presentation; (4) normal fetal well‐being on cardiotocography. Women who had a history of cervical surgery or any contraindication for vaginal delivery were excluded.

### 2.2 Measurement of cervical SWE

**Figure 1:**
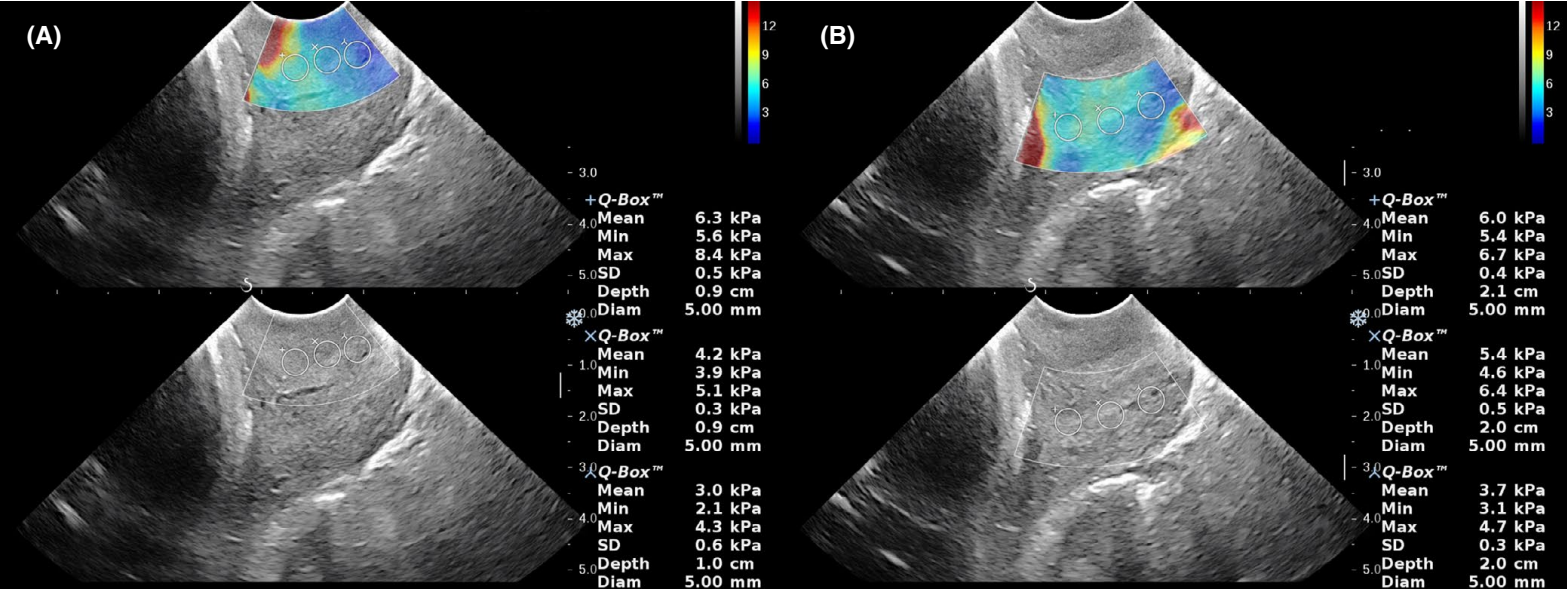
The shear wave elastic measurements. The shear wave elastic measurements were made on the inner, middle and outer parts of the anterior (A) and posterior (B) cervical lip

Participants were assessed on admission for IOL. Before the digital assessment for the BS, women were asked to empty the bladder and in modified lithotomy, a transvaginal scan of the cervix was performed using an SE 12‐3 probe (3‐12 MHz) of the SuperSonic Imagine ultrasound system (Aixplorer supersonic imagine, Aix‐en‐Province, France). The probe was inserted gently without any pressure being exerted on the cervix, and the mid‐sagittal view of the cervix was identified by clear visualization of internal os, canal and external os. The cervical image was magnified to occupy at least 75% of the screen. Once an optimal image of the cervix was obtained, a sampling box was put over the anterior lip and then the posterior lip of the cervix. To optimize the quality of the elastogram color image, each time the size of the sampling box was adjusted to just fit either the anterior or the posterior cervix. Each of the cervical lips was divided into three equal parts along its longitudinal axis: the inner part (proximal one‐third), the middle part, and the outer part (distal one‐third). Then the SWE value of each region of interest (ROI) was measured with a 5‐mm‐diameter circle placed at the center of each ROI. The SWE value was automatically displayed in pressure units (kPa) on the screen (Figure 1). The sampling method was repeated two more times from each cervical lip so as to obtain three independent SWE measurements of each ROI. The average of these three measurements was used for analysis.

The scan were performed by trained ultrasonographers. Their intra‐ and interobserver reproducibility was assessed among the first consecutive 30 women, who were assessed twice by the same operator, and then reassessed by the second operator. All the measurements were made on the independent images selected from the saved images. Both operators were blinded to the measurements made by each other.

### 2.3 Other ultrasonic assessment

**Figure 2:**
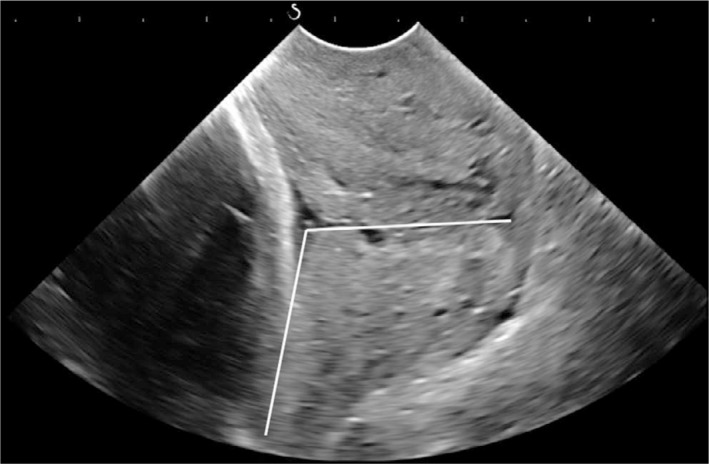
The measurement of the posterior cervical angle, which is the inferior angle between the line joining the internal os and external os, and the line across the lower segment of the posterior uterine wall

**Figure 3:**
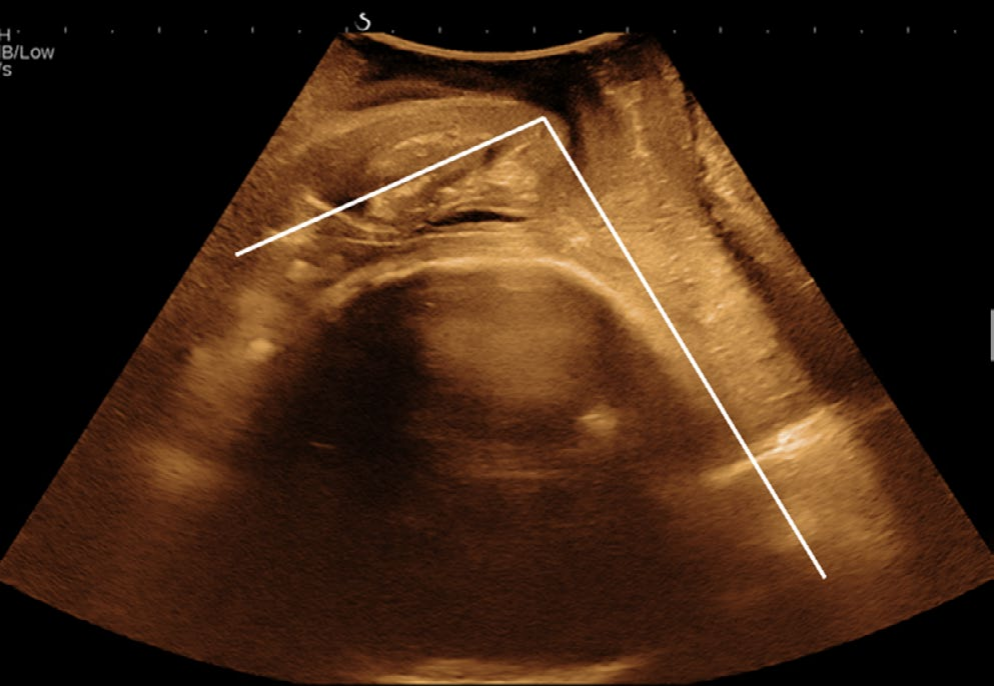
The measurement of the angle of progression, which is the angle between a line crossing the longitudinal axis of pubic symphysis intersecting a line through its inferior point tangential to the outer edge of the fetal skull [Color figure can be viewed at http://wileyonlinelibrary.com]

The cervical length was measured as the linear distance between the internal os and the external os.^16^ The PCA was defined as the inferior angle between the line joining the internal os and external os, and the line across the lower segment of the posterior uterine wall (Figure 2).^5^ A transperineal ultrasound was then performed using a curved XC6‐1 probe (1‐6 MHz) and the AOP was measured on the sagittal view between a line crossing the longitudinal axis of pubic symphysis intersecting a line through its inferior point tangential to the outer edge of the fetal skull (Figure 3).^6^ The pulsatility indices (PI) of the fetal middle cerebral artery (MCA) and the umbilical artery (UA) were also measured and the cerebroplacental ratio (CPR)^17^ was calculated. Estimated fetal weight (EFW) was calculated from the measurements of fetal biometry^18^ and expressed as percentiles.^19^

### 2.4 Management of IOL

Subsequently, an independent obstetrician, blinded to the ultrasound findings, performed the per‐vaginal digital examination to determine the BS. The decision on the method of IOL was based on the BS. The standard practice of the studying unit was that, when the BS was ≥6, the cervix was regarded as ripened or favorable, and the IOL proceeded with amniotomy and/or syntocinon infusion; when the BS was <6, vaginal prostaglandin E2 (PGE2) gel or dinoprostone pessary was used. All clinical staff were blinded to the ultrasound findings. Failure to enter active phase was defined as failure of the cervix to efface and dilate to 3 cm in 12 hours after amniotomy or initiation of syntocinon infusion, or remaining unfavorable (BS <6) in 24 hours after a single pessary of 10 mg dinoprostone or three doses of 3 mg PGE2 gel. Failure to progress in the active phase was defined as cervical dilation slower than 1 cm/h for 4 hours during the active phase of labor. Fetal distress was defined as the presence of pathological cardiotocography which required immediate delivery.^20^

### 2.5 Sample size

Our previous model to predict outcome of induction based on BS alone gave an AUC of 0.65.^7^ To detect a change of 0.1 in AUC with a new prediction model would require a minimum sample size of 425 for a type 1 error of 0.05, 80% power and assuming a CS for failed induction of 20% and a correlation between AUC of 0.5. Planned sample size was increased by a further 10% to 468 to allow for up to 10% failure rate to measure one or more ultrasound markers.

### 2.6 Statistical analyses

The primary outcome of IOL was successful vaginal delivery vs CS. Secondary comparisons were made between the group of vaginal delivery and the subgroups of: (1) CS for failure to enter active phase, (2) CS for failure to progress and (3) CS for fetal distress. The maternal characteristics, fetal and cervical ultrasound parameters were compared using the vaginal delivery group as the reference. Normality of variables was tested using the Kolmogorov–Smirnov test. Normally distributed continuous variables were compared using the Student *t* test, and non‐normal distributed parameters were compared with the Mann‐Whitney *U* test. Categorical variables were compared using the chi‐square test or Fisher exact test as appropriate. The intra‐ and interobserver reproducibility was assessed by the intraclass correlation coefficient (ICC) and Bland‐Altman graphs. SWE values between different cervical regions were compared with paired Wilcoxon signed rank test. A backward stepwise conditional elimination method was used to generate the regression model and to determine the independent predictors for all CS and for CS due to failure to enter active labor.^21^ Receiver‐operating characteristics (ROC) curves were then constructed for the regression models to determine their discriminative ability. The optimal cutoff was determined by the Youden index.^22^ The area under curve (AUC) was compared using the DeLong test. Statistical Package for Social Science (SPSS) version 20.0. (IBM Corp., NY, USA) and medcalc statistical Software version 18 (MedCalc Software bvba, Ostend, Belgium) were used for the statistical analysis. A two‐tailed *P* value <0.05 was considered statistically significant.

### 2.7 Ethical approval

This study was approved by the Joint Chinese University of Hong Kong‐New Territories East Cluster Clinical Research Ethics Committee on 21 April 2015 (CRE: 2015.141). Written informed consent was obtained from all participants.

## 3 RESULTS

A total of 500 pregnant women were recruited, of which 25 were excluded because 12 cases had early sign of spontaneous onset of labor, 4 cases had CS due to suspected macrosomia and 9 cases declined IOL, leaving 475 cases for IOL. The demographic characteristics of the studied population are shown in Table 1. The primary indications for IOL included post‐term pregnancy in 234 (49.3%), gestational diabetes mellitus in 75 (15.8%), suspected macrosomia in 46 (9.7%), oligohydramnios in 28 (5.9%), intrauterine growth restriction in 27 (5.7%), hypertension in 22 (4.6%), history of precipitate labor in 19 (4%), advanced maternal age in 16 (3.4%) and other reasons in 8 (1.7%). Half of the cases (49.9%) needed cervical ripening due to unfavorable cervix. Following IOL, 393 (82.7%) resulted in vaginal delivery and 82 (17.3%) had emergency CS, of which 40 were due to failure to enter active phase, 20 due to fetal distress, 19 due to failure to progress, and three due to other reasons.

**Table 1:** The demographic characteristics of the 475 women who underwent induction of labor

Characteristics	Value
Maternal age (y)	32 (19‐45)
Maternal height (cm)	158 (144‐177)
BMI at delivery (kg/m^2^)	27.34 (19.07‐42.83)
Nulliparous	274 (57.7%)
Gestational age (wk)	40.1 (37‐42)
Bishop score ≥6	238 (50.1%)
Birthweight (g)	3427 (1966‐4195)

Note

Data are given as median (range) or n (%).

Abbreviation: BMI, body mass index.

The ICCs of intra‐ and interobserver reproducibility were >0.85 in each ROI (Table S1, Figures S1 and S2). Table 2 gives the SWE values at each ROI and shows that an elastic gradient exists along the longitudinal axis, with the inner part being significantly stiffer than the middle part, and the middle part being significantly stiffer than the outer part, along both the anterior (5.4 kPa vs 4.8 kPa vs 3.8 kPa; all *P* < 0.001) and posterior lips (5.0 kPa vs 4.7 kPa vs 3.9 kPa; all *P* < 0.001). The SWE values at different ROI are also significantly intercorrelated with each other (Spearman coefficients are shown in Table 3) (all *P* < 0.001). Hence, for subsequent comparison we used the inner cervical SWE (the mean SWE of the inner anterior and inner posterior cervix), the stiffest region.

**Table 2:** Shear wave elastic values at different cervical regions (kPa)

Region	Inner part	Middle part	Outer part	Inner vs Middle	Middle vs Outer
Anterior cervical lip	5.4 (4.3‐6.5)	4.8 (3.8‐5.6)	3.8 (3.1‐4.7)	*P* < 0.001	*P* < 0.001
Posterior cervical lip	5.0 (4.0‐6.0)	4.7 (3.8‐5.7)	3.9 (3.1‐4.7)	*P* < 0.001	*P* < 0.001

Note

Data are given as median (interquartile). The data were compared with paired Wilcoxon signed rank test.

**Table 3:** The Spearman coefficients between shear wave elastic values at different regions

Region	Anterior cervical lip		Posterior cervical lip		
	Middle	Outer	Inner	Middle	Outer
Anterior cervical lip					
Inner	0.77	0.566	0.633	0.509	0.41
Middle	—	0.7	0.548	0.539	0.419
Outer	—	—	0.544	0.536	0.57
Posterior cervical lip					
Inner	—	—	—	0.781	0.618
Middle	—	—	—	—	0.649

Note

The Spearman correlation was performed. All *P *< 0.001.

**Figure 4:**
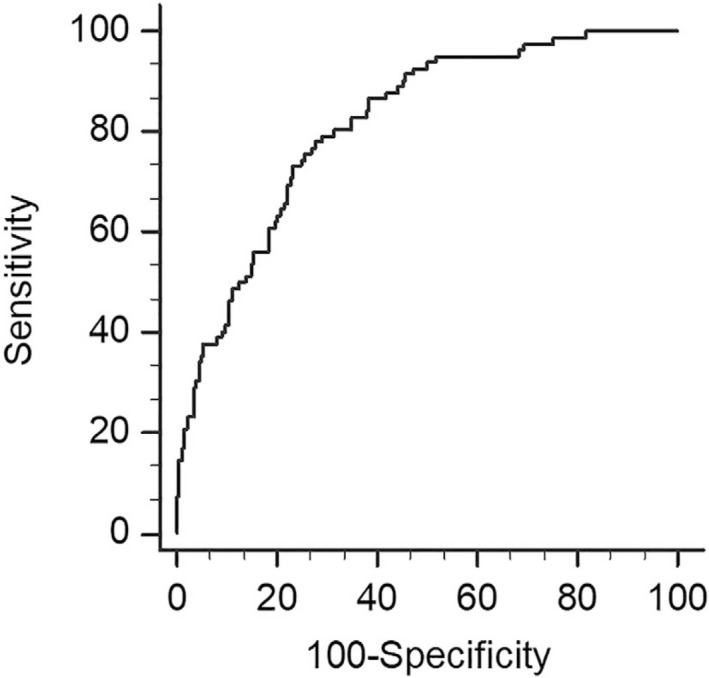
The prediction of all cesarean deliveries. ROC curve for the prediction of all cesarean deliveries after induction of labor with AUC of 0.815 (95% CI 0.777‐0.85)

Comparison of maternal characteristics, the BS, fetal and cervical sonographic measurements between the vaginal delivery group and the whole group of CS is illustrated in Table 4. Table 5 shows the odds ratio (OR) of variables in the univariate analysis and adjusted odds ratio (AOR) in the multivariate analysis for the prediction of CS. The results indicated that the significant independent predictors, in order of strength, multiparity (AOR 0.102, 95% confidence interval [CI] 0.048‐0.22), cervical length (AOR 1.717, 95% CI 1.077‐1.663), inner cervical elasticity (AOR 1.338, 95% CI 1.001‐1.598) and maternal height (AOR 0.894, 95% CI 0.845‐0.946). Combining these four factors, the AUC for the prediction of CS was 0.815 (95% CI 0.777‐0.85) (Figure 4). Body mass index (BMI) ≥0 kg/m^2^, Bishop score and AOP were not independent predictors.

**Table 4:** The comparison of maternal characteristics, Bishop score, fetal and cervical sonographic measurements between the vaginal delivery group and cesarean group

Factors	Vaginal delivery (n = 393)	cesarean group (n = 82)	*P*
Maternal age (≥35 y)^a^	139 (35.4%)	40 (48.8%)	0.023
Maternal height (cm)^b^	158 (155‐162)	155 (152‐159)	<0.001
BMI at delivery (≥30 kg/m^2^)^a^	80 (20.4%)	30 (36.6%)	0.002
Multiparous^a^	192 (48.9%)	9 (11%)	<0.001
Bishop score^b^	6 (4‐6)	3 (3‐4.5)	<0.001
EFW (g)^b^	3325 (3073‐3566)	3556 (3095‐3783)	0.013
EFW <10th centile^a^	28 (7.1%)	8 (9.8%)	0.395
UA PI^c^	0.77 (.67‐.87)	0.76 (.67‐.85)	0.662
MCA PI^c^	1.35 (1.1‐1.59)	1.35 (1.08‐1.51)	0.681
CPR^c^	1.75 (1.45‐2.09)	1.73 (1.41‐2.14)	0.946
Cervical length (cm)^c^	2.4 (1.6‐3.0)	2.9 (2.2‐3.5)	<0.001
Posterior cervical angle (°)^c^	112 (98‐125)	109 (91‐124)	0.04
Angle of progression (°)^c^	87 (80‐96)	84 (75‐88)	<0.001
Inner cervical SWE (kPa)^c^	5.1 (4.2‐6.0)	5.8 (4.9‐7.0)	<0.001

Data are given as median (interquartile range) or n (%).

Abbreviations: BMI, body mass index; CPR, cerebroplacental ratio; EFW, estimated fetal weight; inner cervical SWE, mean of shear wave elasticity of anterior and posterior inner cervix; MCA, middle cerebral artery; PI, pulsatility index; UA, umbilical artery.

a Chi‐square test or Fisher exact test as appropriate.

b Mann‐Whitney *U* test.

c Student *t* test.

**Table 5:** Univariate analysis and multivariate analysis for prediction of cesarean delivery

Variables	Univariate analysis		Multivariate analysis	
	Odds ratio (95% CI)	*P*	Adjusted odds ratio (95% CI)	*P*
Maternal height	0.907 (.864‐.952)	<0.001	0.894 (0.845‐0.946)	<0.001
BMI ≥30 kg/m^2^	2.257 (1.353‐3.767)	0.002	—	—
Multiparous	0.129 (.063‐.265)	<0.001	0.102 (0.048‐0.22)	<0.001
EFW	1.001 (1.000‐1.001)	0.023	—	—
Bishop score	0.605 (.515‐.71)	<0.001	—	—
Cervical length	1.916 (1.451‐2.530)	<0.001	1.717 (1.183‐2.492)	0.004
Angle of progression	0.953 (.931‐.974)	<0.001	—	—
Inner cervical SWE	1.43 (1.214‐1.684)	<0.001	1.338 (1.077‐1.663)	0.009

Binary logistic regression was performed.

Equation 1: Log_e _(odds) = 13.686 – 2.279*parity (0 for nulliparous, 1 for multiparous)‐ 0.112*mat height + 0.541*cervical length + 0.291* inner cervical SWE.

Abbreviations: BMI, body mass index; EFW, estimated fetal weight; inner cervical SWE, mean of shear wave elasticity of anterior and posterior inner cervix.

**Figure 5:**
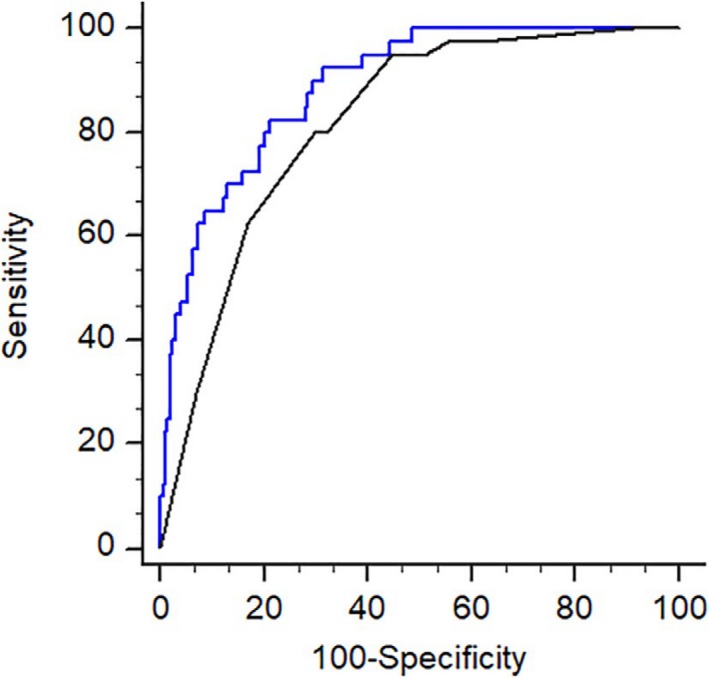
The prediction of cesarean section for failure to enter active phase. ROC curves compare the predictive ability of parity with ultrasonographic measurement (cervical length with inner cervical SWE, black line: AUC 0.888 (95% C: 0.853‐0.916) and parity with Bishop score (dashed line: AUC 0.819 (95% CI 0.778‐0.855) (*P* = 0.009). The diagnostic odds ratio is 17.41 (sensitivity of 82.5% and specificity of 78.7%) and 9.65 (sensitivity of 80% and specificity of 70.7%), respectively [Color figure can be viewed at http://wileyonlinelibrary.com]

**Figure 6:**
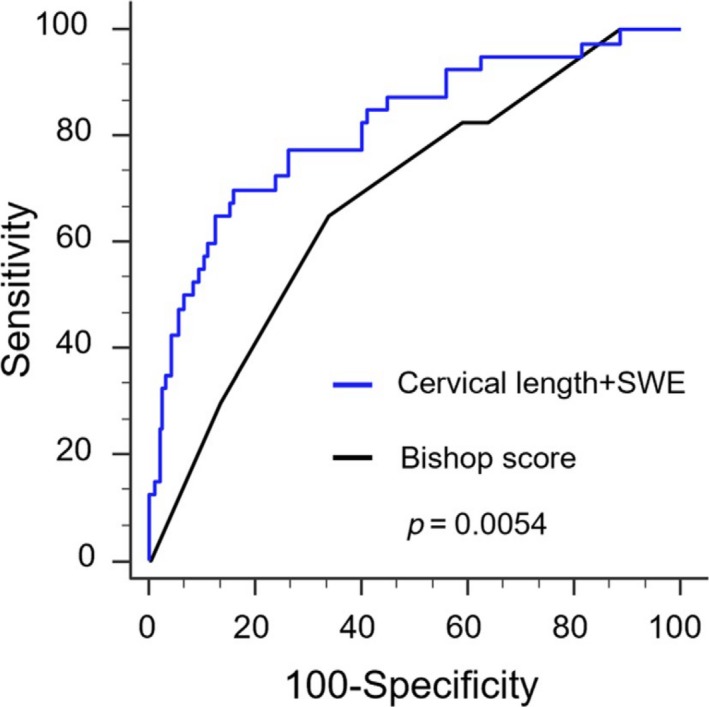
The prediction of cesarean section for failure to enter active phase among nulliparous women. ROC curves compare the predictive ability of sonographic measurement (cervical length with inner cervical SWE (black line: AUC 0.816, 95% CI 0.759‐0.864) and Bishop score (dashed line: AUC 0.68, 95% CI 0.615‐0.74) (*P* = 0.0054). The diagnostic odds ratio is 12.34 (sensitivity of 70.0% and specificity of 84.1%) and 3.80 (sensitivity of 65.0% and specificity of 67.2%), respectively [Color figure can be viewed at http://wileyonlinelibrary.com]

Table 6 shows the comparison of maternal characteristics, and fetal ultrasound parameters and cervical measurements between the group of vaginal delivery and the three subgroups of CS for different indications. Compared with the group of vaginal delivery, women who had CS for failure to enter active phase had a significantly higher inner cervical SWE value (median 6.9 kPa vs 5.1 kPa; *P* < 0.001), as well as a longer cervix, smaller AOP and PCA. However, none of these sonographic parameters was different when comparing the vaginal delivery group with either the subgroup of CS for fetal distress (5.2 kPa) or the subgroup of CS for failure to progress in the active phase (5.0 kPa). Women who failed to enter active phase were also significantly shorter, more obese, having a lower prevalence of multiparity, lower Bishop score, a higher EFW by univariate analysis (Table 6). After multivariate analysis, only parity, cervical length and inner cervical SWE were independent predictors for failure to enter active phase (Table 7), with an AUC of 0.888 (95% CI 0.853‐0.916; Figure 5). If the two cervical ultrasound measurements were replaced by the Bishop score, the AUC significantly dropped to 0.819 (95% CI 0.778‐0.855). The difference between two AUCs was 0.0687 with a 95% CI of 0.0175‐0.12 (DeLong test: *z* = 2.631, *P* = 0.009). As the multi‐parity is the most significant predictor for success of IOL, we further focused on the nulliparous subgroup, and found that sonographic prediction was even stronger than BS among nulliparous women (AUC 0.816, 95% CI 0.759‐0.864 vs 0.68, 95% CI 0.615‐0.74; *P* = 0.0054) (Figure 6). The sensitivity, specificity, positive predictive value, negative predictive value, positive likelihood ratio and negative likelihood ratio of the regression models in all women and in the nulliparous subgroup are shown in Table 8.

**Table 6:** The comparison of maternal characteristics, Bishop score, fetal and cervical ultrasonic measurements between the vaginal delivery group and different groups of cesarean section

Factors	Vaginal delivery (reference) (n = 393)	cesarean for failure to enter active phase		cesarean for failure to progress in active phase		cesarean for fetal distress	
		(n = 40)	*P*	(n = 19)	*P*	(n = 20)	*P*
Maternal age (≥35 y)^a^	139 (35.4%)	18 (45%)	0.227	9 (47.4%)	0.287	11 (55%)	0.075
Maternal height (cm)^b^	158 (155‐162)	156 (154‐160)	0.062	154 (152‐158)	0.002	153 (150‐159)	<0.001
BMI at IOL (≥30 kg/m^2^)^a^	80 (20.4%)	16 (40.0%)	0.004	6 (31.6%)	0.25	6 (30.0%)	0.394
Multiparous^a^	192 (48.9%)	2 (5%)	<0.001	3 (15.8%)	0.005	3 (15%)	0.003
Bishop score^b^	6 (4‐6)	3 (2‐4)	<0.001	4 (3‐5)	0.003	4 (3‐6)	0.028
EFW (g)^b^	3325 (3073‐3566)	3527 (3276‐3770)	0.008	3641 (3511‐3826)	<0.001	3082 (2871‐3498)	0.111
EFW <10th centile^a^	26 (7.0%)	1 (2.5%)	0.498	0	0.617	7 (36.8%)	<0.001
UA PI^c^	0.77 (0.67‐0.87)	0.75 (0.66‐0.85)	0.521	0.79 (0.70‐0.85)	0.75	0.76 (0.67‐0.80)	0.558
MCA PI^c^	1.35 (1.10‐1.59)	1.39 (1.22‐1.66)	0.087	1.16 (1.03‐1.47)	0.084	1.17 (.99‐1.40)	0.02
CPR^c^	1.76 (1.45‐2.09)	1.89 (1.56‐2.21)	0.051	1.47 (1.27‐1.93)	0.093	1.64 (1.29‐2.09)	0.19
Cervical length (cm)^c^	2.4 (1.6‐3.0)	3.3 (2.7‐3.7)	<0.001	2.5 (1.6‐3.0)	0.795	2.4 (2.1‐3.5)	0.122
Posterior cervical angle (°)^c^	112 (98‐125)	102 (84‐121)	0.018	121 (106‐128)	0.6	110 (95‐126)	0.786
Angle of progression (°)^c^	87 (80‐96)	81 (73‐88)	<0.001	86 (82‐88)	0.081	85 (79‐86)	0.101
Inner cervical SWE^c^	5.1 (4.2‐6.0)	6.9 (5.5‐7.6)	<0.001	5.0 (4.6‐5.7)	0.937	5.2 (4.6‐5.9)	0.793

All the comparisons were made with in the vaginal delivery group. Data are given as median (interquartile range) or n (%).

Abbreviations: BMI, body mass index; CPR, cerebroplacental ratio; EFW, estimated fetal weight; MCA, middle cerebral artery; PI, pulsatility index; UA, umbilical artery.

a Chi‐square test or Fisher exact test as appropriate.

b Mann‐Whitney *U* test.

c Student *t* test.

**Table 7:** Univariate analysis and multivariate analysis for prediction of cesarean delivery for failure to enter active phase

Variables	Univariate analysis		Multivariate analysis	
	Odds ratio (95% CI)	*P*	Adjusted odds ratio (95% CI)	*P*
Multiparous	0.055 (0.013‐0.232)	<0.001	0.029 (0.006‐0.142)	<0.001
Bishop score	0.52 (0.411‐0.658)	<0.001	—	—
Cervical length	3.019 (1.981‐4.602)	<0.001	2.556 (1.462‐4.467)	0.001
Angle of progression	0.942 (0.914‐0.971)	<0.001	—	—
Inner cervical SWE	1.825 (1.448‐2.299)	<0.001	1.689 (1.234‐2.311)	0.001

Binary logistic regression was performed.

Equation 2: Log_e _(odds) = −7.228 – 3.533*parity (0 for nulliparous, 1 for multiparous) + 0938*cervical length + 0.524* inner cervical SWE.

Abbreviation: SWE, shear wave elastography.

**Table 8:** The screening performance of different predictors of failure to enter active labor

Predictor	AUC (95% CI)	Cutoff	Sensitivity	Specificity	PPV	NPV	+LR	−LR
Parity + CL + inner cervical SWE	0.888 (0.853‐.916)	>0.1031	82.5%	78.7%	29.2%	97.7%	3.88	0.22
Parity + Bishop score	0.819 (0.778‐.855)	>0.0989	80.0%	70.7%	21.8%	97.2%	2.73	0.28
Nulliparity								
CL + inner cervical SWE	0.816 (0.759‐.864)	>0.2247	7.0%	84.1%	38.4%	92.3%	4.4	0.36
Bishop score	0.680 (0.615‐.740)	>0.1601	65.0%	67.2%	28.3%	9.6%	1.98	0.52

Receiver‐operating characteristics curves were constructed and the Youden index was used to determine the optimal cutoff.

Abbreviations: AUC, area under curve; CL, cervical length; inner cervical SWE, mean of shear wave elasticity of anterior and posterior inner cervix; LR, likelihood ratio; NPV, negative predictive value; PPV, positive predictive value.

In the subgroup of CS indicated for failure to progress in the active phase, EFW was significantly higher and the Bishop score lower than in the vaginal delivery group. The BMI was also higher and maternal height less, and there were fewer multiparous women. In the subgroup of CS for fetal distress, the MCA PI were significantly lower, the proportion with EFW below 10th percentile was higher, the mothers were shorter and there were fewer multiparous women; the BS was also lower, all based on univariate analysis (Table 6).

## 4 DISCUSSION

This is the first study using SWE to predict the outcome of IOL, and it demonstrated that (1) the stiffness of the cervix decreases towards the outer cervix; (2) the inner cervical SWE and cervical length are independent predictors for overall CS, as well as for the subgroup of CS indicated for failure to enter active phase, but AOP, PCA and the BS are not; (3) a model using the combination of cervical length, inner cervical SWE, parity and maternal height, can achieve an AUC of .815 in the prediction of overall CS after IOL; and, with the former three factors, an AUC of .888 for CS for failure to enter active phase.

Our finding of decreasing stiffness from the inner to the outer part of the cervix is concordant with several studies that have shown the spatial heterogeneity in the stiffness within the cervix using SWE.^15^ This has been hypothesized to be attributable to the cervical collagen fiber orientation.^23^ The collagen cross‐link around the internal os is significantly more heterogeneous than that around the external os, and therefore the stroma around the internal os functions distinctively from the external os.^23^ Hernandez‐Andrade et al found that the stiffness of the inner cervix is more predictive of spontaneous preterm delivery.^24^ They showed that a hard internal os at 16‐24 weeks is 80% less likely to have spontaneous preterm delivery compared with a soft internal os.^24^ In a small cohort study, a hard internal os was associated with the failure of IOL.^10^ Therefore, the inner cervical SWE was selected in the regression analysis. Besides the objective measurement of the cervical stiffness, SWE also has a potential advantage over manual examination, as the latter cannot easily access the innermost part of the cervix.

In contrast to previous studies, our study showed that SWE is useful in the prediction of overall CS following IOL. So far, only a few small‐scale studies have attempted to evaluate elastography in predicting the outcomes of IOL, and their results are controversial.^10^, ^11^, ^12^, ^13^ Pereira et al^11^ concluded that elastography is not useful in predicting the IOL outcome. However, our study differs from Pereira's in many significant ways. First of all, they used a semiquantitative strain‐based elastography, which relies on the internal organ movement, whereas the shear wave elastography we used has the advantage of quantifying the cervical stiffness independent of adjacent tissues and operators’ movements or internal organ movement. Secondly, they focused on a small spot at the internal os of the canal, which is the gland or sometimes the mucus, but we surveyed the cervical elasticity on the stroma, which contributes to the mechanical strength^23^ and then selected the stiffest inner cervix as the reference.^25^ Thirdly, they recruited only 99 pregnant women, whereas the sample size of our cohort is five times larger. A recent meta‐analysis^26^ combined the findings of four small‐cohort studies of a total of 323 subjects,^12^, ^13^, ^27^, ^28^ and suggested that strain‐based elastography might be predictive of successful IOL. However, the reported AUC of cervical elastography was only 0.55, which was no better than that of the BS (0.51) and much poorer than that of cervical length (0.70). Our findings are superior to the meta‐analysis in several ways. First, the heterogeneity among the reviewed studies could impact the power of the meta‐analysis. Secondly, we were able to show that both cervical elastography and cervical length are independent factors, whereas BS is not. Finally, we achieved a relatively high AUC of 0.815 and 0.888, respectively, for the prediction of overall CS after IOL and that of the subgroup requiring CS for failure to enter active phase of labor. Even after excluding multi‐parity, which is the strongest predictor of IOL outcome, and focus on nulliparous women, the combination of cervical elastography and cervical length was even better than BS with the difference of 0.136 between two AUCs

We also found that PCA and AOP, which are respectively the proxies of cervical position and fetal head station in the BS, are no longer independent predictors when SWE is included. This result provides further evidence of intercorrelation between the different components of the BS.^29^ As shown in our comparison of the regression models (Figure 5, Table 8), sonographic measurement of cervical length and SWE is superior to manual assessment of the BS in predicting failure to enter active phase. Transvaginal ultrasonic examination also causes less pain than digital examination.^30^ However, SWE is not yet readily available in routine ultrasound machine, and it is also expensive to purchase such a machine with cutting‐edge technology.

Our subgroup analysis showed that the SWE and other sonographic parameters are not useful in predicting CS indicated due to fetal distress or failure to progress. This is biologically understandable, as fetal distress is unrelated to cervical favorability but rather restricted fetal growth and fetal compromise, whereas failure to progress is related more to large fetal size, as reflected from our results (Table 6). Our findings also indicate that it is not straightforward to create a prediction model for all CS. Whereas a large fetal weight increases the chance of CS for slow progress, a small fetus is associated with CS for fetal distress. The effect of fetal weight may be masked when overall CS is the primary outcome.

The major strengths of our study are that, by measuring elasticity in different regions of the cervix, we demonstrated that the inner part of the cervix is the most useful predictor of different regions of the cervix. The large sample size from a homogeneous ethnic group is another advantage of our study. However, the overall number of CS of 80 can only allow a maximum of eight variables for multivariate analysis. Therefore we could only select the eight strongest variables based on univariate analysis.^21^ Nonetheless, our study has tested multiple clinical and ultrasonic variables, of which the combination has significantly improved the prediction compared with using clinical variables alone.^31^ The choice of the method of IOL was based on the BS alone. It is worth investigating in future research whether SWE may provide a better guide of IOL method and improve the chances of success.

## 5 CONCLUSION

Shear wave elastography is a useful tool in pre‐IOL assessment of the stiffness of the cervix, which is an independent predictor of overall CS, and specifically CS indicated for the failure to enter active phase. PCA, AOP and the Bishop score were not independent predictors of CS. The combination of sonographic cervical length and shear‐wave elastography is superior to the Bishop score in predicting failure of IOL.

## CONFLICT OF INTEREST

The authors have stated explicitly that there are no conflicts of interest in connection with this article.

## References

[1] Rayburn WF , Zhang J . Riding rates of labor induction: present concerns and future strategies. Obstet Gynecol. 2002;100:164‐167. 12100818 10.1016/s0029-7844(02)02047-1

[2] Bishop EH . Pelvic scoring for elective induction. Obstet Gynecol. 1964;24:266‐268. 14199536

[3] Faltin‐traub EF , Boulvain M , Faltin DL , Extermann P , Irion O . Reliability of the Bishop score before labour induction at term. Eur J Obstet Gynaecol Reprod Biol. 2004;112:178‐181. 10.1016/s0301-2115(03)00336-1 14746954

[4] Kolkman DG , Verhoeven CJ , Brinkhorst SJ , et al. The Bishop score as a predictor of labor induction success: a systematic review. Am J Perinatol. 2013;30:625‐630. 23283806 10.1055/s-0032-1331024

[5] Rane SM , Guirgis RR , Higgins B , Nicolaides KH . The value of ultrasound in the prediction of successful induction of labor. Ultrasound Obstet Gynecol. 2004;24:538‐549. 15386612 10.1002/uog.1100

[6] Barbera AF , Pombar X , Peruginoj G , Lezotte DC , Hobbins JC . A new method to assess fetal head descent in labor with transperineal ultrasound. Ultrasound Obstet Gynecol. 2009;33:313‐319. 19248000 10.1002/uog.6329

[7] Cheung CW , Leung TY , Sahota DS , et al. Outcome of induction of labour using maternal characteristics, ultrasound assessment and biochemical state of the cervix. J Matern Neonatal Med. 2010;23:1406‐1412. 10.3109/14767051003678135 20230317

[8] Gillor M , Vaisbuch E , Zaks S , Barak O , Hagay Z , Levy R . Transperineal sonographic assessment of the angle of progression as a predictor of a successful vaginal delivery following induction of labor. Ultrasound Obstet Gynecol. 2017;49:240‐245. 27062415 10.1002/uog.15931

[9] Molina FS , Gómez LF , Florido J , Padilla MC , Nicolaides KH . Quantification of cervical elastography: a reproducibility study. Ultrasound Obstet Gynecol. 2012;39:685‐689. 22173854 10.1002/uog.11067

[10] Swiatkowska‐Freund M , Preis K . Elastography of the uterine cervix: implications for success of induction of labor. Ultrasound Obstet Gynecol. 2011;38:52‐56. 21484905 10.1002/uog.9021

[11] Pereira S , Frick AP , Poon LC , Zamprakou A , Nicolaides KH . Successful induction of labor: prediction by preinduction cervical length, angle of progression and cervical elastography. Ultrasound Obstet Gynecol. 2014;44:468‐475. 24832011 10.1002/uog.13411

[12] Muscatello A , Di Nicola M , Accurti V , et al. Sonoelastography as method for preliminary evaluation of uterine cervix to predict success of induction of labor. Fetal Diagn Ther. 2014;35:57‐61. 24247111 10.1159/000355084

[13] Hee L , Rasmussen CK , Schlütter JM , Sandager P , Uldbjerg N . Quantitative sonoelastography of the uterine cervix prior to induction of labor as a predictor of cervical dilation time. Acta Obstet Gynecol Scand. 2014;93:684‐690. 24702544 10.1111/aogs.12389

[14] Doherty J , Trahey G , Nightingale K , Palmeri M . Acoustic radiation force elasticity imaging in diagnostic ultrasound. IEEE Trans Ultrason Ferroelectr Freq Control. 2013;60:685‐701. 23549529 10.1109/TUFFC.2013.2617 PMC3679553

[15] Peralta L , Molina FS , Melchor J , et al. Transient elastography to assess the cervical ripening during pregnancy: a preliminary study. Ultraschall Med. 2017;38:395‐402. 26251994 10.1055/s-0035-1553325

[16] Markham KB , Iams JD . Measuring the cervical length. Clin Obstet Gynecol. 2016;59:252‐263. 27042799 10.1097/GRF.0000000000000204

[17] Mari G , Hanif F , Kruger M , Cosmi E , Santolaya‐Forgas J , Treadwell MC . Middle cerebral artery peak systolic velocity: a new Doppler parameter in the assessment of growth‐restricted fetuses. Ultrasound Obstet Gynecol. 2007;29:310‐316. 17318946 10.1002/uog.3953

[18] Hadlock FP , Harrist RB , Sharman RS , Deter RL , Park SK . Estimation of fetal weight with the use of head, body, and femur measurements—a prospective study. Am J Obstet Gynecol. 1985;151:333‐337. 3881966 10.1016/0002-9378(85)90298-4

[19] Cheng YKY , Lu J , Leung TY , Chan YM , Sahota DS . Prospective assessment of INTERGROWTH‐21 st and World Health Organization estimated fetal weight reference curves. Ultrasound Obstet Gynecol. 2018;51:792‐798. 28452092 10.1002/uog.17514

[20] Committee on Obstetric Practice, American College of Obstetricians and Gynecologists . ACOG Committee Opinion. Number 326, December 2005. Inappropriate use of the terms fetal distress and birth asphyxia. Obstet Gynecol. 2005;106:1469‐1470. 16319282 10.1097/00006250-200512000-00056

[21] Peduzzi P , Concato J , Kemper E , Holford TR , Feinstein AR . A simulation study of the number of events per variable in logistic regression analysis. J Clin Epidemiol. 1996;49:1373‐1379. 8970487 10.1016/s0895-4356(96)00236-3

[22] Zweig MH , Campbell G . Receiver operator characteristic (ROC) plots: a fundamental evaluation tool in clinical medicine. Clin Chem. 1993;39:561‐577. 8472349

[23] Zork NM , Myers KM , Yoshida K , et al. A systematic evaluation of collagen cross‐links in the human cervix. Am J Obstet Gynecol. 2015;212:321.e1‐321.e8. 25281365 10.1016/j.ajog.2014.09.036 PMC4346506

[24] Hernandez‐Andrade E , Romero R , Korzeniewski SJ , et al. Cervical strain determined by ultrasound elastography and its association with spontaneous preterm delivery. J Perinat Med. 2014;42:159‐169. 24356388 10.1515/jpm-2013-0277 PMC4183449

[25] Yao W , Gan Y , Myers KM , Vink JY , Wapner RJ , Hendon CP . Collagen fiber orientation and dispersion in the upper cervix of non‐pregnant and pregnant women. PLoS ONE. 2016;29:e0166709. 10.1371/journal.pone.0166709 PMC5127549 27898677

[26] Londero AP , Schmitz R , Bertozzi S , Driul L , Fruscalzo A . Diagnostic accuracy of cervical elastography in predicting labor induction success: a systematic review and meta‐analysis. J Perinat Med. 2016;44:167‐178. 26011923 10.1515/jpm-2015-0035

[27] Hwang HS , Sohn IS , Kwon HS . Imaging analysis of cervical elastography for prediction of successful induction of labor at term. J Ultrasound Med. 2013;32:937‐946. 23716514 10.7863/ultra.32.6.937

[28] Fruscalzo A , Londero AP , Fröhlich C , Möllmann U , Schmitz R . Quantitative elastography for cervical stiffness assessment during pregnancy. Biomed Res Int. 2014;2014:826535. 24734246 10.1155/2014/826535 PMC3964773

[29] Williams MC , Krammer J , O'Brien WF . The value of the cervical score in predicting successful outcome of labor induction. Obstet Gynecol. 1997;90:784‐789. 9351765 10.1016/S0029-7844(97)00415-8

[30] Tan PC , Vallikkannu N , Suguna S , Quek KF , Hassan J . Transvaginal sonographic measurement of cervical length vs. Bishop score in labor induction at term: tolerability and prediction of cesarean delivery. Ultrasound Obstet Gynecol. 2007;29:568‐573. 17444553 10.1002/uog.4018

[31] Levine LD , Downes KL , Parry S , Elovitz MA , Sammel MD , Srinivas SK . A validated calculator to estimate risk of cesarean after an induction of labor with an unfavorable cervix. Am J Obstet Gynecol. 2018;218:254.e1‐254.e7. 29224730 10.1016/j.ajog.2017.11.603 PMC5807156

