# Supplementary figures and images for: The predictive value of cervical shear wave elastography in the outcome of labor induction
Source: Acta Obstet Gynecol Scand. 2019 Nov 5;99(1):59–68. doi: 10.1111/aogs.13706 (PMC6973099; doi:10.1111/aogs.13706)

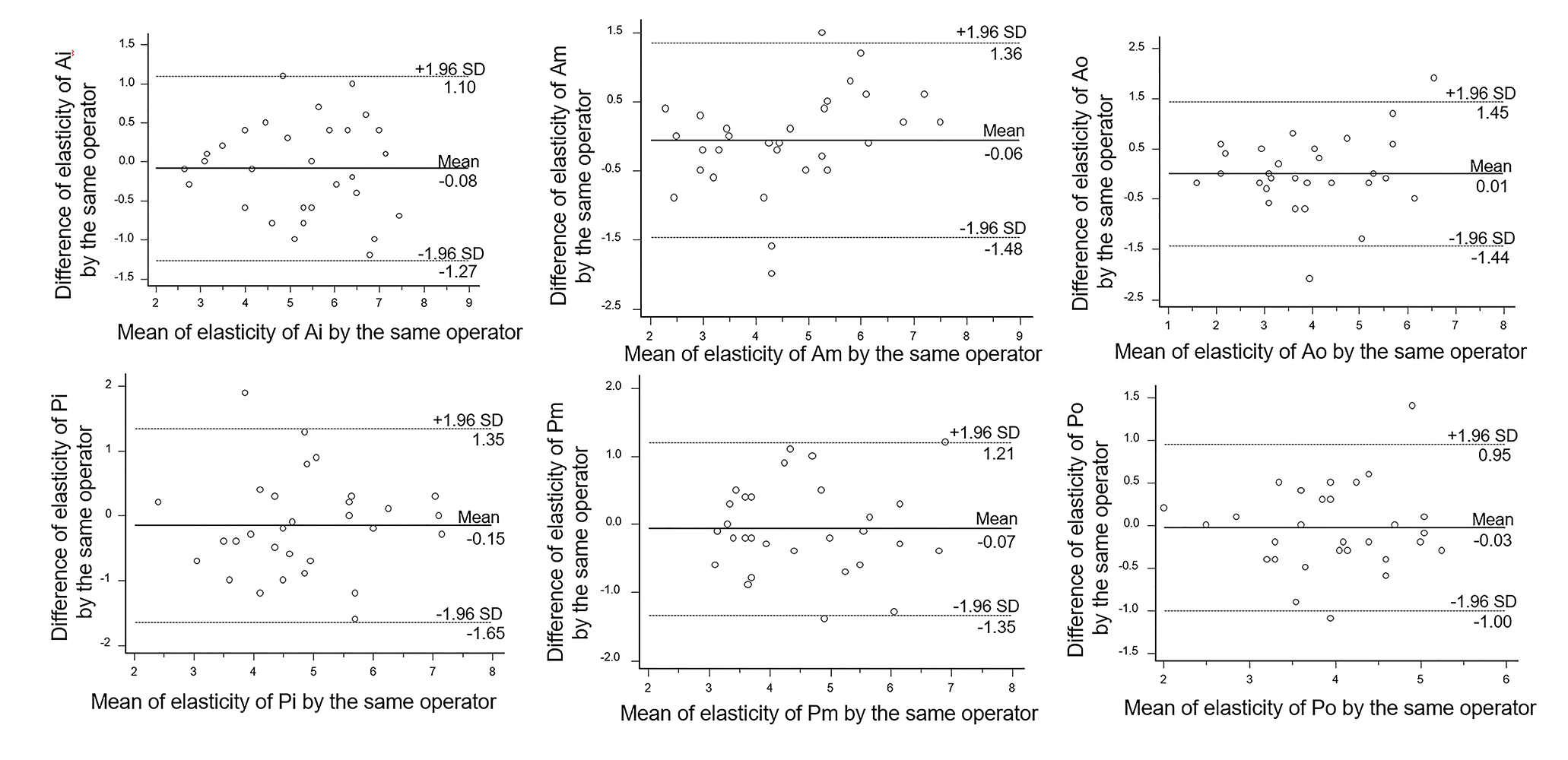

Supplement: Supplementary file 1 [file AOGS-99-59-s001.tif]

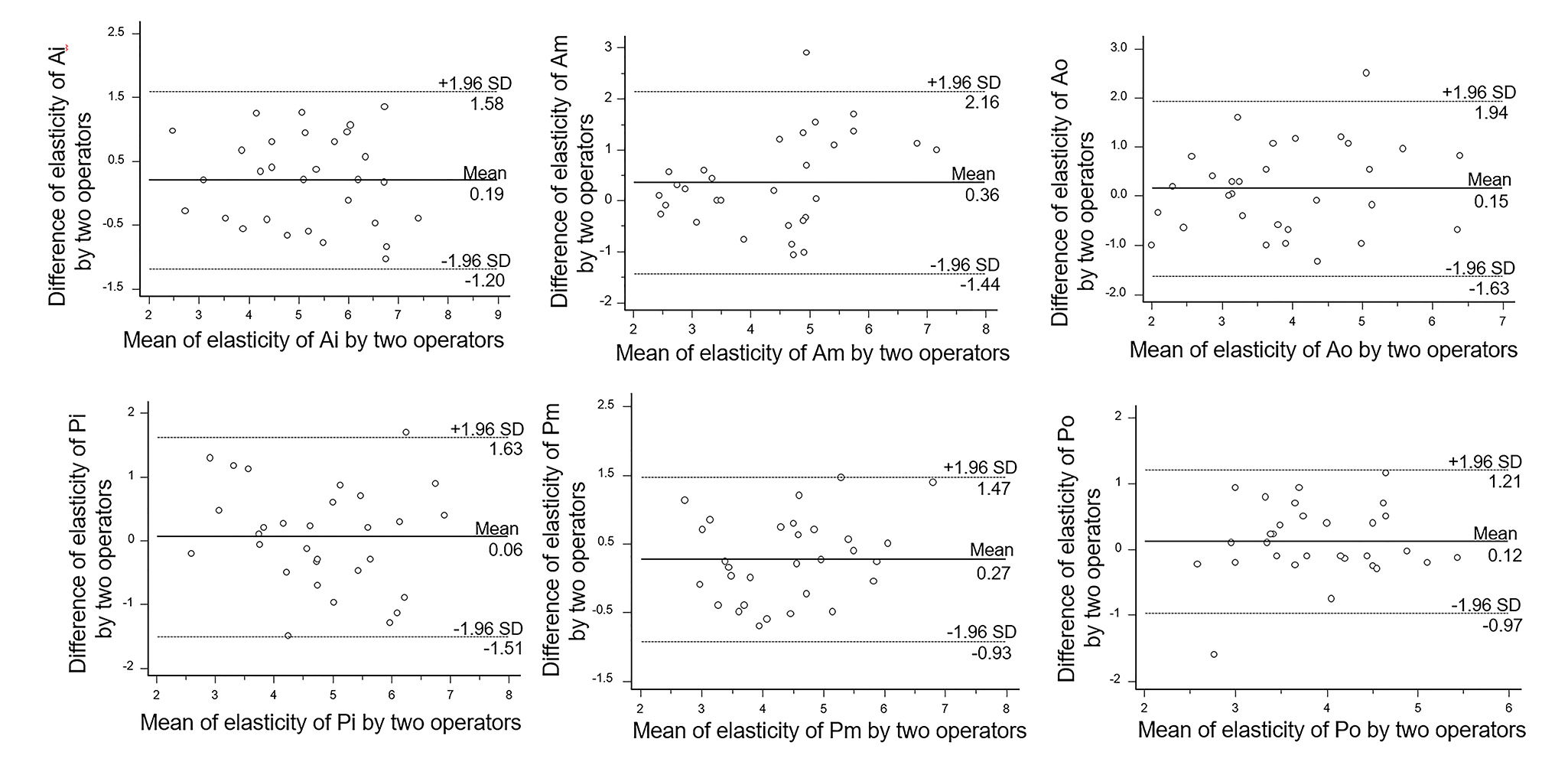

Supplement: Supplementary file 2 [file AOGS-99-59-s002.tif]
