# Supplementary material for: The predictive value of cervical shear wave elastography in the outcome of labor induction
Source: Acta Obstet Gynecol Scand. 2019 Nov 5;99(1):59–68. doi: 10.1111/aogs.13706 (PMC6973099; doi:10.1111/aogs.13706)
Supplement: Supplementary file 3 [file AOGS-99-59-s003.docx]

Intra-class correlation coefficients of intra- and inter-observer reproducibility

| ROI | Intra-observer  reproducibility (95%CI) | *p* | Inter-observer  reproducibility (95%CI) | *p* |
| --- | --- | --- | --- | --- |
| Anterior cervical lip | |  |  |  |
| Inner | 0.954 (0.903-0.978) | <0.001 | 0.922 (0.838-0.963) | <0.001 |
| Middle | 0.938 (0.869-0.970) | <0.001 | 0.856 (0.689-0.932) | <0.001 |
| Outer | 0.920 (0.832-0.962) | <0.001 | 0.850 (0.687-0.928) | <0.001 |
| Posterior cervical lip | |  |  |  |
| Inner | 0.892 (0.775-0.948) | <0.001 | 0.884 (0.756-0.945) | <0.001 |
| Middle | 0.922 (0.837-0.963) | <0.001 | 0.900 (0.770-0.954) | <0.001 |
| Outer | 0.901 (0.792-0.953) | <0.001 | 0.852 (0.692-0.929) | <0.001 |

**Supporting Information legends**

Table S1. Intra-class correlation coefficients of intra- and inter-observer reproducibility at six locations.

Figure S1. Bland-Altman graphs for the intra-observer reproducibility of SWE measurements at six cervical locations.

Figure S2. Bland-Altman graphs for the inter-observer reproducibility of SWE measurements at six cervical locations.
